# Characterization of the Receptors for Mycobacterial Cord Factor in Guinea Pig

**DOI:** 10.1371/journal.pone.0088747

**Published:** 2014-02-12

**Authors:** Kenji Toyonaga, Yasunobu Miyake, Sho Yamasaki

**Affiliations:** 1 Division of Molecular Immunology, Medical Institute of Bioregulation, Kyushu University, Fukuoka, Japan; 2 Research Center for Advanced Immunology, Medical Institute of Bioregulation, Kyushu University, Fukuoka, Japan; National Institute of Infectious Diseases, Japan

## Abstract

Guinea pig is a widely used animal for research and development of tuberculosis vaccines, since its pathological disease process is similar to that present in humans. We have previously reported that two C-type lectin receptors, Mincle (macrophage inducible C-type lectin, also called Clec4e) and MCL (macrophage C-type lectin, also called Clec4d), recognize the mycobacterial cord factor, trehalose-6,6′-dimycolate (TDM). Here, we characterized the function of the guinea pig homologue of Mincle (gpMincle) and MCL (gpMCL). gpMincle directly bound to TDM and transduced an activating signal through ITAM-bearing adaptor molecule, FcRγ. Whereas, gpMCL lacked C-terminus and failed to bind to TDM. mRNA expression of gpMincle was detected in the spleen, lymph nodes and peritoneal macrophages and it was strongly up-regulated upon stimulation of zymosan and TDM. The surface expression of gpMincle was detected on activated macrophages by a newly established monoclonal antibody that also possesses a blocking activity. This antibody potently suppressed TNF production in BCG-infected macrophages. Collectively, gpMincle is the TDM receptor in the guinea pig and TDM-Mincle axis is involved in host immune responses against mycobacteria.

## Introduction

Tuberculosis is a life-threatening disease caused by the infection of *Mycobacterium tuberculosis* (*M. tuberculosis*) and related strains. About one-third of the world's population are suffering from this infectious disease. At present, *M. bovis* bacillus Calmette-Guerin (BCG) is the only available vaccine against tuberculosis, however, its effectiveness is still controversial for adults [1]–[3]. This has led to an urgent need for development of a new tuberculosis vaccine. To that end, it is necessary to understand the molecular mechanisms of recognition and activation of tuberculosis by the host immune system.

Mycobacteria contain a wide variety of components that elicit the host immune system. Trehalose-6,6′-dimycolate (TDM), also called the cord factor, has been demonstrated to be the most potent stimulator of inflammatory responses among the *M. bovis* BCG cell wall glycolipids [4]. In addition, injection of pure TDM into mice causes the formation of lung granulomas that are a characteristic feature of tuberculosis patients [5]. We have previously reported that C-type lectin Mincle (macrophage inducible C-type lectin, also called Clec4e) and MCL (macrophage C-type lectin, also called Clec4d) recognize TDM and transduce an activating signals through ITAM-bearing adaptor molecule, FcRγ[6], [7]. Mincle is an essential receptor for TDM-induced innate immune responses such as granulomagenesis, and macrophage activation because these responses are almost completely abolished in Mincle-deficient mice [6], [8].

Animal models have been used for the research and development of new vaccines for tuberculosis [9], [10]. Guinea pig is highly sensitive to *M. tuberculosis* infection and a low-dose of aerosol infection causes pulmonary tuberculosis that shares important morphological and clinical features with human tuberculosis [11]–[13]. However, the majority of infectious experiments of tuberculosis have been carried out in mouse models because of the limited availability of study tools for guinea pigs.

In this study, we report that gpMincle but not gpMCL acts as a TDM receptor. gpMincle mRNA is preferentially expressed in lymphoid organs and myeloid cells. gpMincle protein was expressed in activated macrophages and functioned as an FcRγ-coupled activating receptor for TDM. We further established an anti-gpMincle blocking antibody.

## Materials and Methods

### Reagents

TDM (T3034) and zymosan (Z4250) were purchased from Sigma-Aldrich. *M. tuberculosis* H37Ra and *M. bovis* BCG were obtained from Difco and Japan BCG Laboratory, respectively. ELISA kit for guinea pig TNF (DY5035) was from R&D Systems. For stimulation of reporter cells and peritoneal macrophages, TDM dissolved in chloroform:methanol (2∶1) at 1 mg/ml were diluted in isopropanol and added on 96-well plates at 20 µl/well, followed by evaporation of the solvent as previously described [6].

### Antibodies

The monoclonal antibody (mAb) to gpMincle (5H4) was established by immunization of Mincle^−/−^ mice [14] with T cell hybridoma cells (2B4) expressing gpMincle. Anti-mMincle mAb (4A9) and anti-hMincle mAb (13D10-H11) were described elsewhere [15], [16]. Anti-HA mAbs clone HA124 and TANA2 were from Nacalai tesque and MBL, respectively. Anti-HA polyclonal antibody (pAb) (sc-805) was from SantaCruz. Anti-HA pAb (ab72564) was from abcam. Anti-Flag pAb (F7425) was from Sigma-Aldrich.

### Experimental Animals

Two or three-week-old female outbred strain Hartley guinea pigs were purchased from Kyudo. Animal protocols were approved by the committee of Ethics on Animal Experiment, Faculty of Medical Sciences, Kyushu University.

### Cells

2B4-NFAT-GFP reporter cells expressing Mincle or MCL together with FcRγ were established as previously described [7], [14], [15], [17]. Bronchoalveolar lavage (BAL) cells were isolated from BAL fluid. BAL was performed by tracheal cannulation and washing the lung with RPMI 1640 medium. Peripheral blood cells were isolated from heparinized blood. Bone marrow cells were obtained by flushing femora with RPMI 1640 medium. Debris was removed by cell strainer and red blood cells were lysed by ammonium chloride solution. Peritoneal macrophages were obtained as follows: guinea pigs were injected intraperitoneally with 20 ml of 3% thioglycollate medium (Difco). Four days after the injection, peritoneal exudate cells were harvested. After removal of the contaminating tissue debris by cell strainer, red blood cells in the cell suspension were lysed with ammonium chloride solution. Cells were cultured in DMEM medium supplemented with 10% FBS for 2 h. Non-adherent cells were removed and the adherent cells were collected and used for experiments.

### Real-time PCR

cDNA from various tissues (brain, colon, heart, kidney, liver, lung, skeletal muscle, small intestine, stomach) was purchased from BioChain. Total RNA from tissues and cells was isolated by Sepasol-RNA I Super G (Nacalai tesque). Real-time PCR was performed by using THUNDERBIRD SYBR qPCR Mix (TOYOBO) and ABI PRISM 7000 (Applied Biosystems). The primer sets for gpMincle was sense (5′-ACCTCCAfTCCTGCTTCTCACC-3′) and anti-sense (5′-GTTGCCAATTCAGTGGACAGC-3′), and gpβ-actin was sense (5′-TGCTGCGTTACACCCTTTCTTG-3′) and anti-sense (5′-CAACCAACTGCTGTCACCTTCA-3′).

### Ig-fusion proteins

Mincle-Ig fusion proteins were prepared as described previously [7], [15], [16]. In brief, C-terminus of the extracellular domains of gpMincle (45-210 a.a.), mMincle (46-214 a.a.) and hMincle (45-219 a.a.) were fused to N-terminus of human IgG1 Fc region (gpMincle-Ig, mMincle-Ig and hMincle-Ig, respectively). Ig-fusion proteins were incubated with 200 ng/well of plate-coated TDM, and bound proteins were detected by using HRP-labeled anti-human IgG (Jackson ImmunoResearch).

### Cloning of guinea pig Mincle, MCL and FcRγ

Full length cDNA of gpMincle, gpMCL and gpFcRγ was generated by PCR using the following primer pairs; for gpMincle, 5′-AAGGATCCCACCATGAATTCTTCCCAATCTTG-3′ (sense) and 5′-GCGGCCGCGTGTGGTTATGCTTG-3′ (anti-sense); for gpMCL, 5′-TTGAAT TCATGCAACTAGTAGAACC-3′ (sense) and 5′-GTACTCGAGTTACTTGAG CACTGTTCCAGG-3′ (anti-sense); for gpFcRγ, 5′-TTGAATTCATGTATCCAG CAGTGG-3′ (sense) and 5′-GCGGCCGCTTACTGGGGCGGTTTC-3′ (anti-sense).

### Constructions

gpMincle and gpMCL cDNA fragments were cloned by PCR and inserted into pMX-IRES-human CD8 vectors. Flag or HA tags were fused at the C-terminus of gpMincle and gpMCL by PCR. gpFcRγ cDNA fragment was cloned by PCR and inserted into pMX-IRES-rat CD2 or pMX-IRES-GFP vectors. gpFcRγ (19-86 a.a.) was fused to the C-terminus of Flag tag and inserted into the downstream of human CD8 signal peptide.

### Statistics

An unpaired two-tailed Student's *t* test was used for all the statistical analyses.

## Results

### Expression of Mincle mRNA in guinea pig tissue

**Figure 1:**
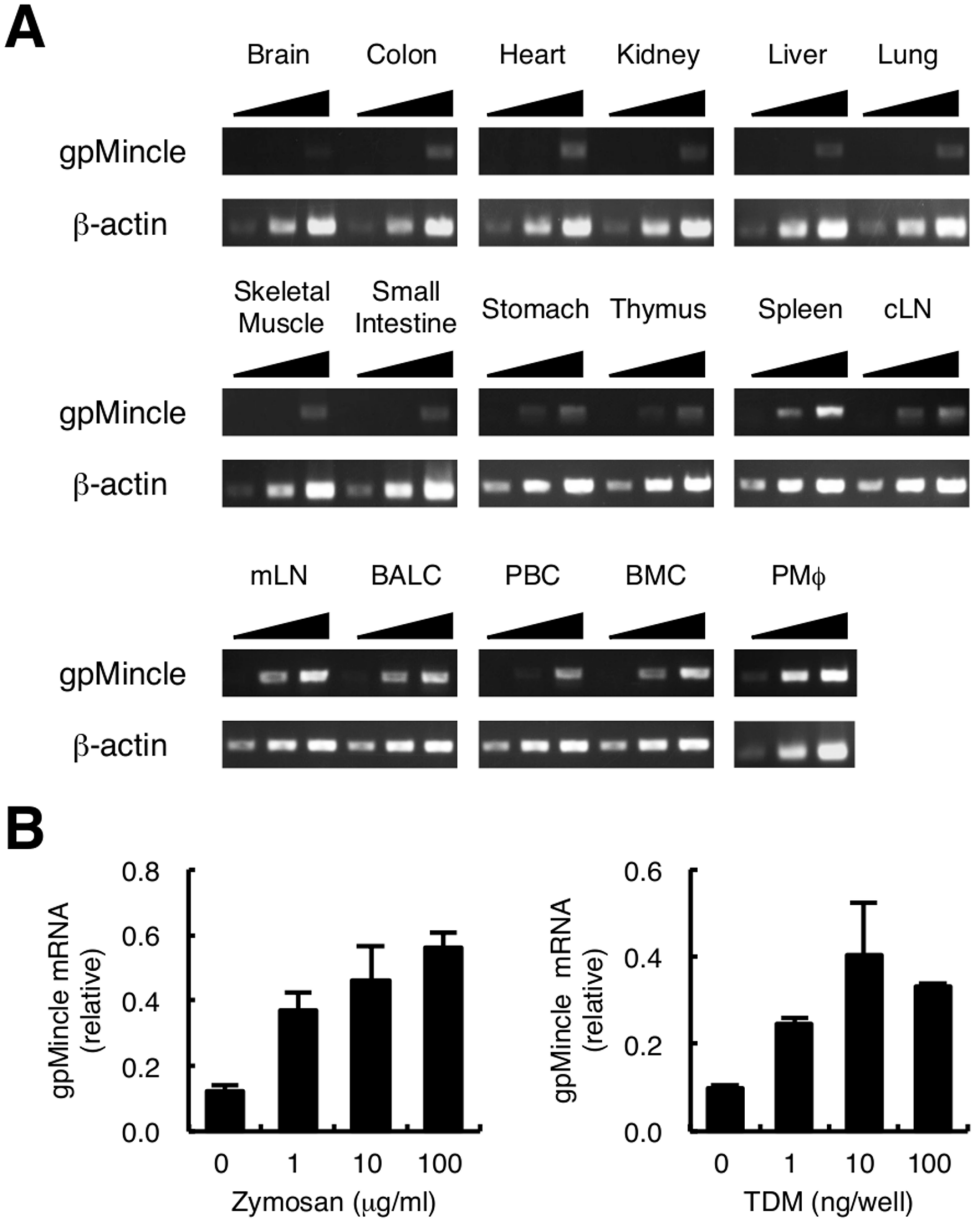
Expression of gpMincle mRNA. (A) Tissue distribution of gpMincle mRNA. mRNA expression of gpMincle in indicated tissues (cLN, cutaneous lymph node; mLN, mesenteric lymph node) and cells (BALC, bronchoalveolar lavage cell; PBC, peripheral blood cell; BMC, bone marrow cell; PMø, thioglycollate-elicited peritoneal macrophage) was detected by PCR. PCR was performed by increased cycle numbers (20, 24, 28 for β-actin and 32, 36, 40 for Mincle). (B) gpMincle mRNA is induced upon stimulation. Macrophages were stimulated with indicated concentrations of zymosan (left panel) or TDM (right panel). mRNA expression of gpMincle was analyzed by RT-PCR at 8 h after stimulation. Data are presented as mean ± s.d. (B) and representative of two separate experiments.

We first analyzed the tissue distribution of Mincle mRNA in guinea pigs by PCR using gene-specific primers (Fig. 1A). gpMincle mRNA was detected in the lymphoid organs; such as, spleen, cutaneous (inguinal) lymph nodes and mesenteric lymph nodes. A higher expression was observed in myeloid cells, including thioglycollate-elicited peritoneal macrophages and bronchoalveolar lavage cells that contain mainly alveolar macrophages. Mincle is an inducible gene and is strongly up-regulated upon cell stimulation [7], [15], [18]. It has been reported that guinea pig macrophages can be activated by typical pathogen-associated molecular patterns (PAMPs) such as zymosan and TDM [19], [20]. We therefore stimulated peritoneal macrophages with these PAMPs and found that the mRNA expression of gpMincle was enhanced upon stimulation (Fig. 1B). Collectively, tissue distribution and inducible expression pattern of gpMincle were comparable to human and murine Mincle.

### gpMincle is an FcRγ-coupled TDM receptor

**Figure 2:**
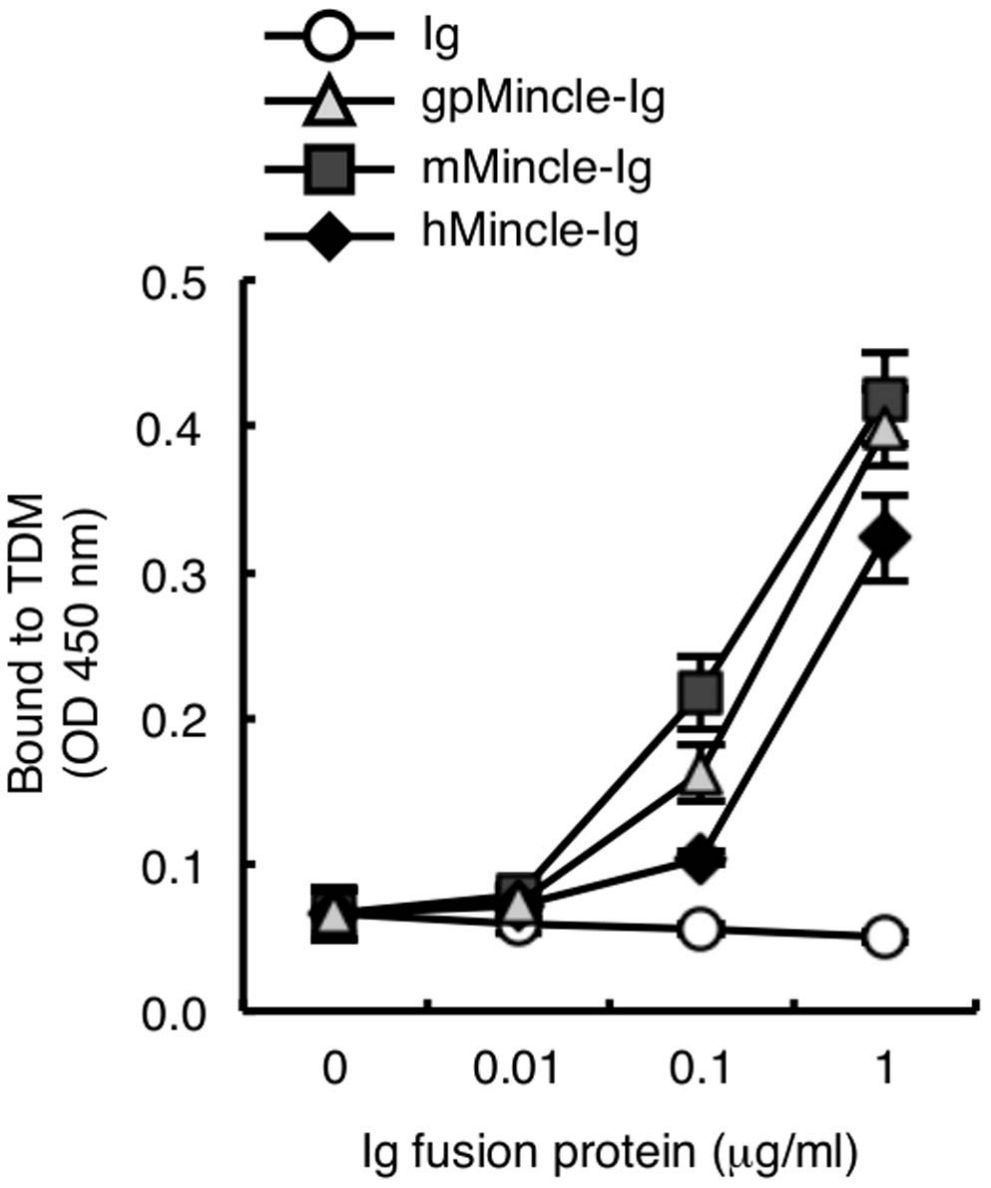
gpMincle directly binds to TDM. Ig-fusion proteins of guinea pig Mincle (gpMincle-Ig), mouse Mincle (mMincle-Ig) and human Mincle (hMincle-Ig) were incubated with plate-coated TDM. Bound proteins were detected with anti-hIgG-HRP. Data are presented as mean ± s.d. and representative of two separate experiments.

The recognition of TDM by mouse and human Mincle requires the Glu-Pro-Asn (EPN) sequence [6], [16], which is a putative mannose-binding motif within the carbohydrate recognition domain (CRD) [21]. Since this EPN motif is present in gpMincle (Fig. S1A black box), we examined whether gpMincle also recognizes TDM. gpMincle cDNA was cloned from the mRNA of peritoneal macrophages obtained from outbred strain guinea pig Hartley, the most widely used strain for tuberculosis analysis in guinea pigs. To prepare a soluble form of gpMincle, the extracellular domain of gpMincle was fused to the carboxyl terminus of the human IgG Fc domain (gpMincle-Ig). gpMincle-Ig as well as mMincle-Ig and hMincle-Ig bound to plate-coated TDM (Fig. 2). This result indicates that gpMincle recognizes TDM directly.

**Figure 3:**
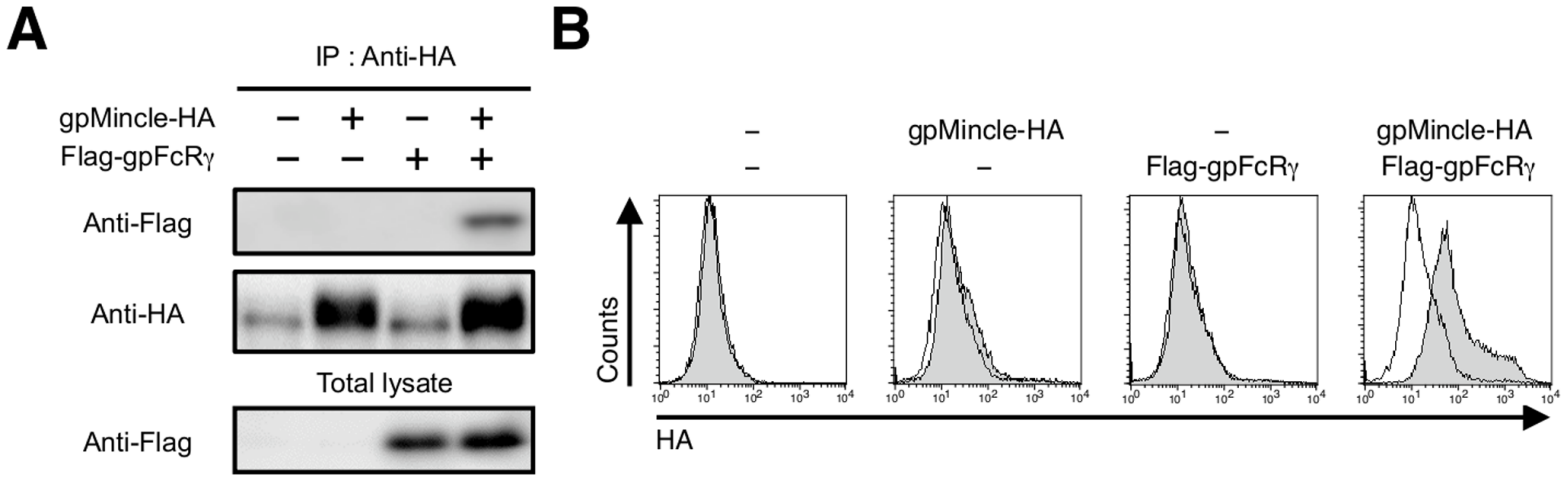
gpMincle is associated with FcRγ. (A) Interaction of gpMincle with gpFcRγ. HEK293T cells were transfected with HA-tagged gpMincle alone or together with Flag-tagged gpFcRγ Total lysates were immunoprecipitated with anti-HA mAb and blotted with anti-Flag and anti-HA polyclonal antibodies (pAbs). Total lysates were also blotted with anti-Flag pAb. (B) Surface expression of gpMincle. HEK293T cells were transfected with gpMincle-HA alone or together with Flag-gpFcRγ. Surface expression of gpMincle was detected by anti-HA pAb. Data are presented as representative of two separate experiments.

Mincle is known to be an FcRγ-coupled activating receptor [15]. We therefore investigated whether gpMincle was associated with gpFcRγ, because the positive-charged arginine residue that mediates the interaction with FcRγ in the transmembrane domain was present in gpMincle (Fig. S1A filled circle). HA-tagged gpMincle was transfected into the human embryonic kidney (HEK) 293T cells alone or together with gpFcRγ. An immunoprecipitation analysis showed that gpFcRγ is co-precipitated with gpMincle, indicating the association of gpMincle with gpFcRγ (Fig. 3A). Anti-HA staining of HEK293T cells showed that surface expression of gpMincle was only detectable in the presence of gpFcRγ, suggesting that gpMincle forms a complex with gpFcRγ on the cell surface (Fig. 3B).

**Figure 4:**
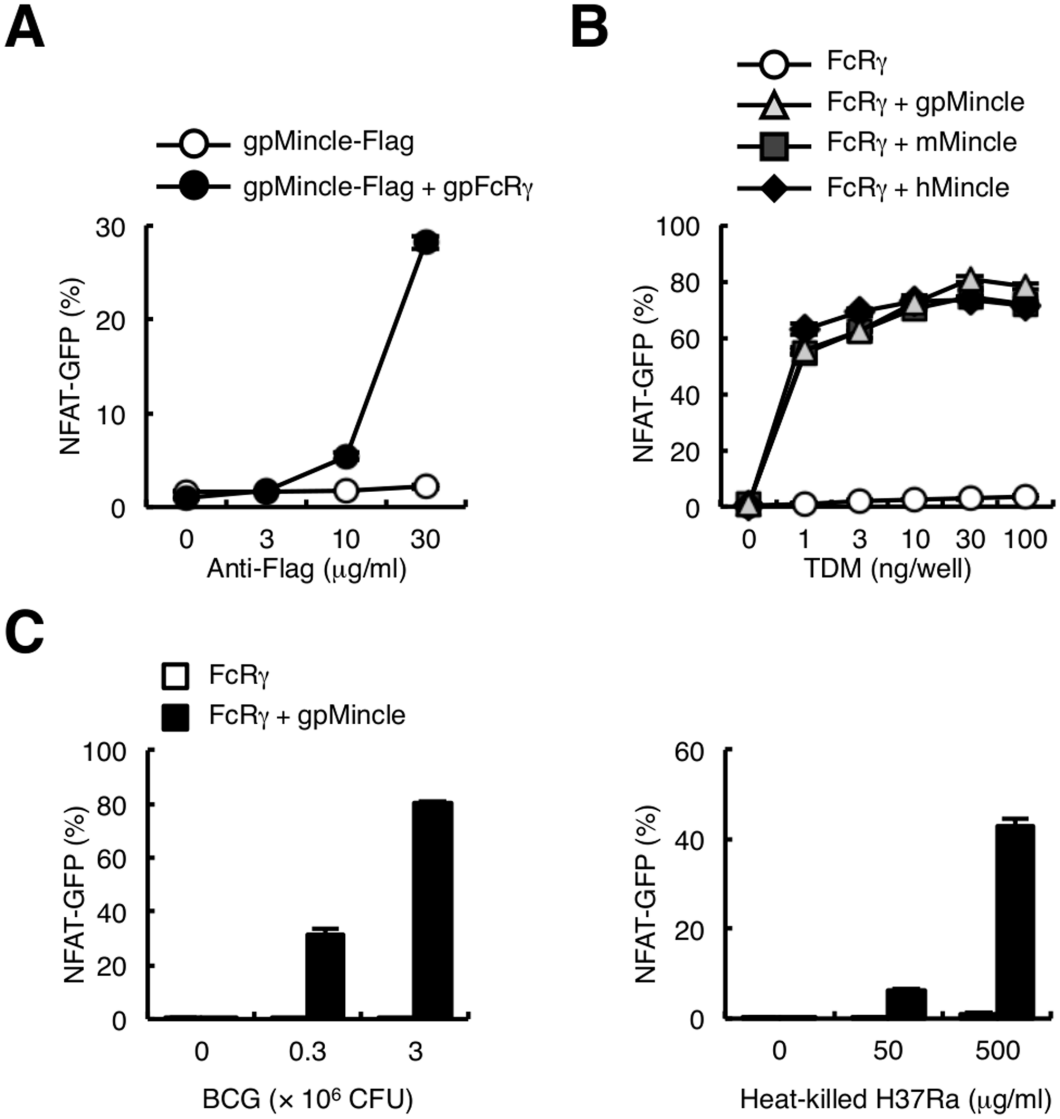
gpMincle functions as an activating receptor for TDM. (A) gpMincle transduces activation signal through gpFcRγ. NFAT-GFP reporter cells were transfected with Flag-tagged gpMincle alone or together with gpFcRγ. Reporter cells were stimulated with plate-coated anti-Flag mAb for 24 h. Induction of NFAT-GFP was analyzed by flow cytometry. (B) gpMincle is a TDM receptor. Indicated reporter cells were stimulated with plate-coated TDM for 24 h. Induction of NFAT-GFP was analyzed by flow cytometry. (C) gpMincle recognizes mycobacteria. Reporter cells were stimulated with *M. bovis* BCG (left panel) and heat-killed *M. tuberculosis* H37Ra (right panel) for 24 h. Induction of NFAT-GFP was analyzed by flow cytometry. Data are presented as mean ± s.d. and representative of two or three separate experiments.

To verify the capacity of gpMincle for transducing signals through FcRγ, Flag-tagged gpMincle alone or together with gpFcRγ was introduced into 2B4 NFAT (nuclear factor of activated T-cells)-GFP reporter cells [7], [14], [15], [17]. In contrast to HEK293T cells, gpMincle was expressed on the cell surface of 2B4 NFAT-GFP reporter cells even in the absence of gpFcRγ and the co-expression of gpFcRγ enhanced the surface expression of gpMincle (data not shown). Plate-coated anti-Flag mAb activated NFAT-GFP only in the presence of gpFcRγ, indicating that gpMincle transduces an activating signal through gpFcRγ (Fig. 4A).

We next examined whether TDM activates gpMincle reporter cells. Because the signal transduction capacity was similar between gpFcRγ and mouse FcRγ in gpMincle reporter cells (data not shown), we used mFcRγ for this experiment to compare gpMincle with mMincle and hMincle. Indeed, the transmembrane domain that is critical for interaction with Mincle possesses a high homology among species (mouse vs. guinea pig, 90% identical; mouse vs. human, 95% identical). TDM strongly activated NFAT-GFP in the reporter cells expressing gpMincle with the same strength as human and mouse Mincle (Fig. 4B). Importantly, *M. bovis* BCG and *M. tuberculosis* H37Ra strongly activated gpMincle reporter cells (Fig. 4C). Collectively, these results indicate that gpMincle recognizes mycobacterial TDM and transduces an activating signal via FcRγ.

### Generation of monoclonal antibody for gpMincle

**Figure 5:**
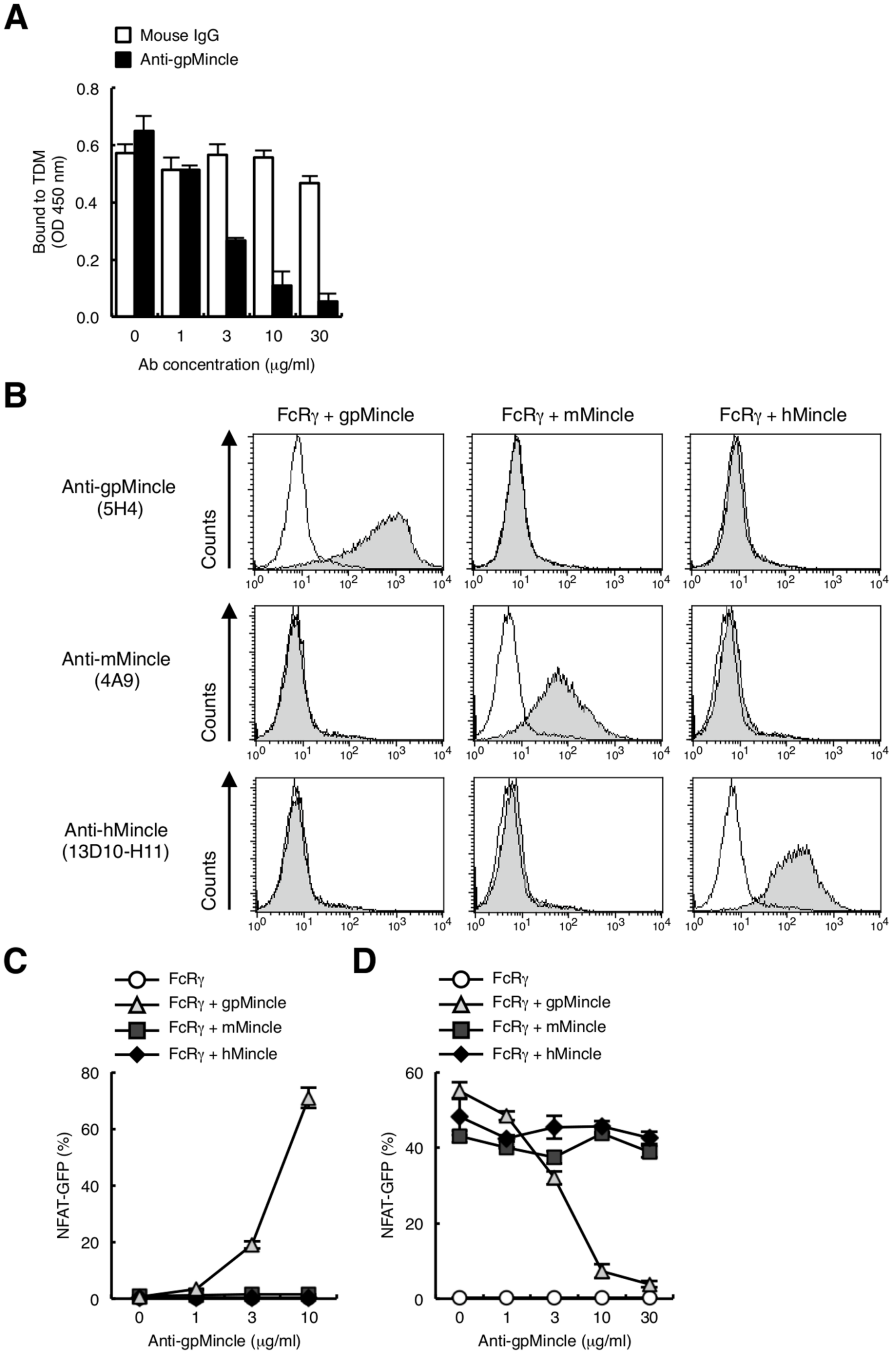
Establishment of anti-gpMincle mAb. (A) Anti-gpMincle mAb blocks interaction of gpMincle-Ig with TDM. gpMincle-Ig (3 µg/ml) were incubated with plate-coated TDM in the presence of anti-gpMincle mAb or mouse IgG. Bound proteins were detected with anti-hIgG-HRP. (B) Surface staining by anti-gpMincle mAb. Indicated reporter cells were stained with anti-gpMincle mAb (5H4, upper panels), anti-mMincle mAb (4A9, middle panels) or anti-hMincle mAb (13D10-H11, lower panels). Open histograms show staining with isotype control IgG. (C) Anti-gpMincle mAb activates NFAT-GFP reporter cells. Indicated reporter cells were stimulated with plate-coated anti-gpMincle mAb for 24 h. Induction of NFAT-GFP was analyzed by flow cytometry. (D) Anti-gpMincle mAb blocks TDM recognition. Indicated reporter cells were treated with anti-gpMincle followed by stimulation with TDM (10 ng/well) for 24 h. Induction of NFAT-GFP was analyzed by flow cytometry. Data are presented as mean ± s.d. (A, C, D) and representative of two or three separate experiments.

To further characterize the function of endogenous gpMincle, we attempted to establish monoclonal antibodies against gpMincle, because an available antibody has not yet been reported. Mincle-deficient mice were immunized with gpMincle-transfected cells to obtain an antibody, since gpMincle shares high homology with mouse Mincle (Fig. S1A). Supernatants of cloned hybridomas were screened for the activity to inhibit the interaction of gpMincle-Ig with TDM. Only one clone 5H4 (IgG1, κ) was obtained (Fig. 5A) and used in this study. Clone 5H4 specifically recognized gpMincle neither mMincle nor hMincle on the surface of transfectant cells (Fig. 5B upper panels). Notably, surface expression of mMincle and hMincle were confirmed by anti-mMincle (Fig. 5B middle panels) and anti-hMincle mAbs (Fig. 5B lower panels), respectively. Given that 5H4 selectively blocked gpMincle–ligand interaction, it is most likely that mAb recognizes a species-specific site within CRD of gpMincle. In spite of its strong recognition of gpMincle on the cell surface, 5H4 failed to react with denatured form of gpMincle by western blotting (data not shown). Taken together, it is possible that 5H4 recognizes conformational epitope of gpMincle.

We then examined whether anti-gpMincle mAb can activate gpMincle-expressing reporter cells. Plate-coated anti-gpMincle mAb potently induced NFAT-GFP through gpMincle but not mMincle or hMincle (Fig. 5C). In addition, pre-treatment of the cells with anti-gpMincle mAb strongly suppressed TDM-induced NFAT activation (Fig. 5D). This antibody did not have any impact on human or mouse Mincle-expressing reporter cells. Taken together, we first established anti-gpMincle specific monoclonal antibody 5H4 that possesses blocking activity.

### Functional analysis of endogenous gpMincle

**Figure 6:**
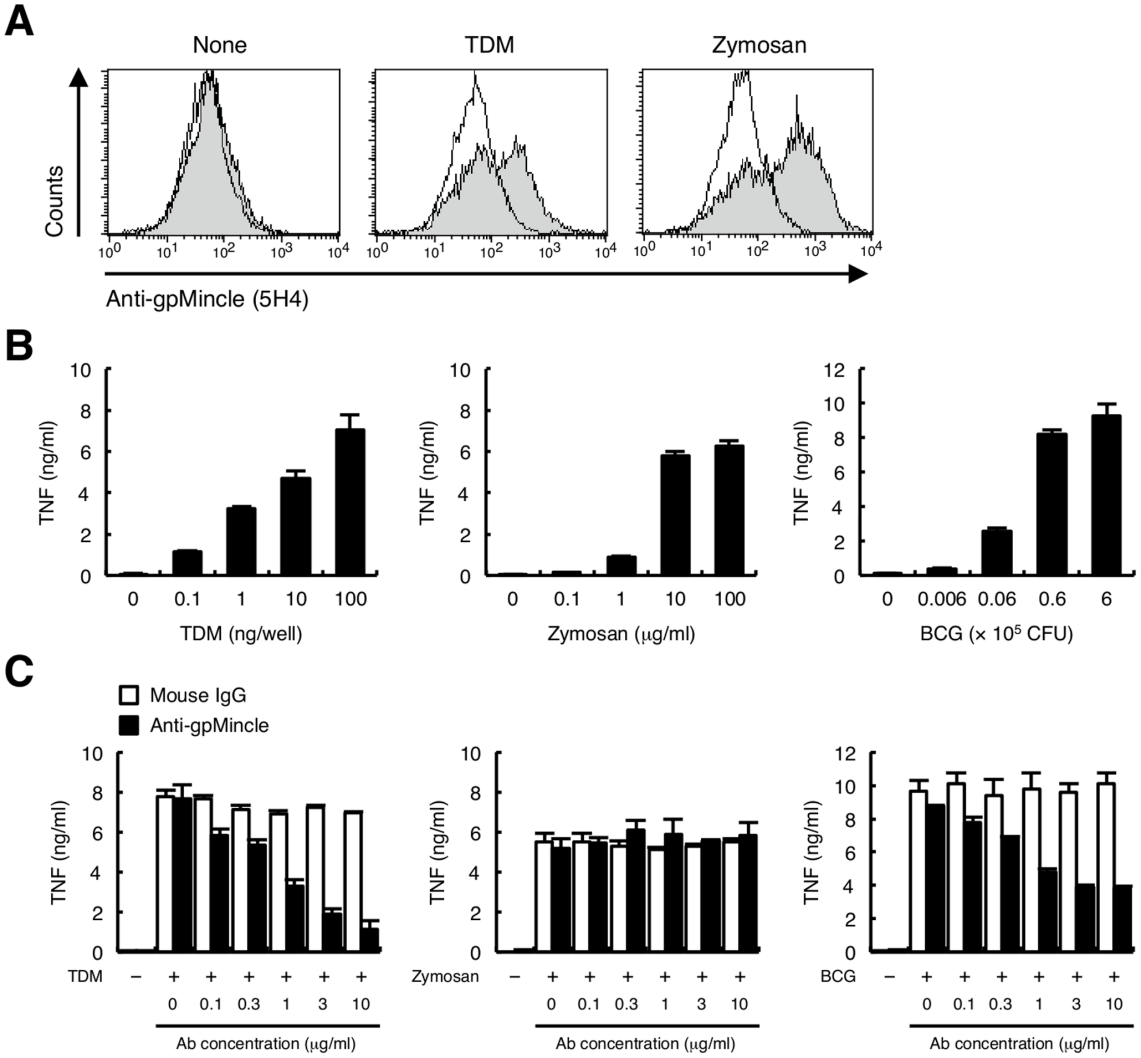
Functional analysis of endogenous gpMincle. (A) Surface expression of gpMincle on macrophage. Macrophages were left untreated (left panel) or stimulated with 10 ng/well TDM (middle panel) or 10 µg/ml zymosan (right panel) for 24 h, and stained with anti-gpMincle mAb (filled histograms) or mouse IgG (open histograms). (B) TNF production upon stimulation. Macrophages were stimulated with TDM (left panel), zymosan (middle panel) or *M. bovis* BCG (right panel) for 48 h. Concentrations of TNF in the culture supernatants were determined by ELISA. (C) Anti-gpMincle mAb blocks TNF production. Macrophages were stimulated with 100 ng/well TDM (left panel), 10 µg/ml zymosan (middle panel) or 6×10^5^ CFU *M. bovis* BCG (right panel) in the presence of anti-gpMincle mAb or mouse IgG for 48 h. Concentrations of TNF in the culture supernatants were determined by ELISA. Data are presented as mean ± s.d. (B, C) and representative of two or three separate experiments.

We finally analyzed the function of endogenous gpMincle by using anti-gpMincle mAb. To detect the endogenous gpMincle, thioglycollate-elicited peritoneal macrophages were stained with anti-gpMincle mAb. gpMincle was barely detectable under non-stimulatory conditions; however, TDM or zymosan stimulation strongly enhanced the surface expression of gpMincle (Fig. 6A). These results are consistent with the mRNA expression of gpMincle, as shown in Figure 1B. We concluded that 5H4 could recognize endogenous gpMincle in freshly isolated cells.

Guinea pig macrophages produced a large amount of pro-inflammatory cytokine, TNF upon stimulation of TDM, zymosan and *M. bovis* BCG (Fig. 6B). Anti-gpMincle mAb strongly suppressed TDM-induced TNF production (Fig. 6C left). In contrast, zymosan stimulation was not affected (Fig. 6C middle). Although *M. bovis* BCG contains a number of stimulatory molecules, anti-gpMincle mAb significantly blocked TNF production from *M. bovis* BCG-infected macrophages (Fig. 6C right). These data suggested that the TDM-Mincle axis is critical for TNF production during BCG infection.

### Functional analysis of gpMCL

Recently, we reported that C-type lectin receptor MCL, which shares a high homology to Mincle, is also critically involved in TDM-mediated immune responses [7]. To obtain the cDNA encoding an MCL homologue from the widely used outbred Hartley guinea pig, we designed the primer pairs encompassing the nucleotide sequence of predicted MCL gene in inbred 2N strain (gpMCL^2N^) from public NCBI database (XM_003470566). The amino acid sequence deduced from the obtained cDNA (gpMCL^Ha^) showed overall similarity to gpMCL^2N^, hMCL, rMCL and mMCL (Fig. S1B asterisks). However, C-terminal region of gpMCL^Ha^ showed low homology with those of other species and it lacked the WND (Trp-Asn-Asp) sequence that is crucial for calcium binding (Fig. S1B gpMCL^Ha^). In addition, gpMCL^Ha^ lacked a large portion of C-terminal end that is supposed to be critical for ligand binding. According to the reported sequence, gpMCL^2N^ contains a premature stop codon in the CRD, which results in the truncation of C-terminal region including WND sequence (Fig. S1B gpMCL^2N^). However, nucleotide acid sequence after the stop codon represented 44 codon stretch corresponding to the conserved amino acid sequence of MCL proteins (Fig. S1C gray). gpMCL^Ha^ cDNA had a two base pair insertion in the coding region of gpMCL^2N^ (Fig. S1C red), which causes frame shift mutation generating stop codon at position 197 (Fig. S1B gpMCL^Ha^). Thus, the C-terminal portions of gpMCL^Ha^ and gpMCL^2N^ have been deleted during evolution through independent genetic mechanism, i.e. an insertion for frame shift and a substitution for stop codon, respectively. Collectively, these results suggest that MCL has lost its functional CRD in guinea pigs. Hereafter, we used gpMCL^Ha^ for further experiments.

**Figure 7:**
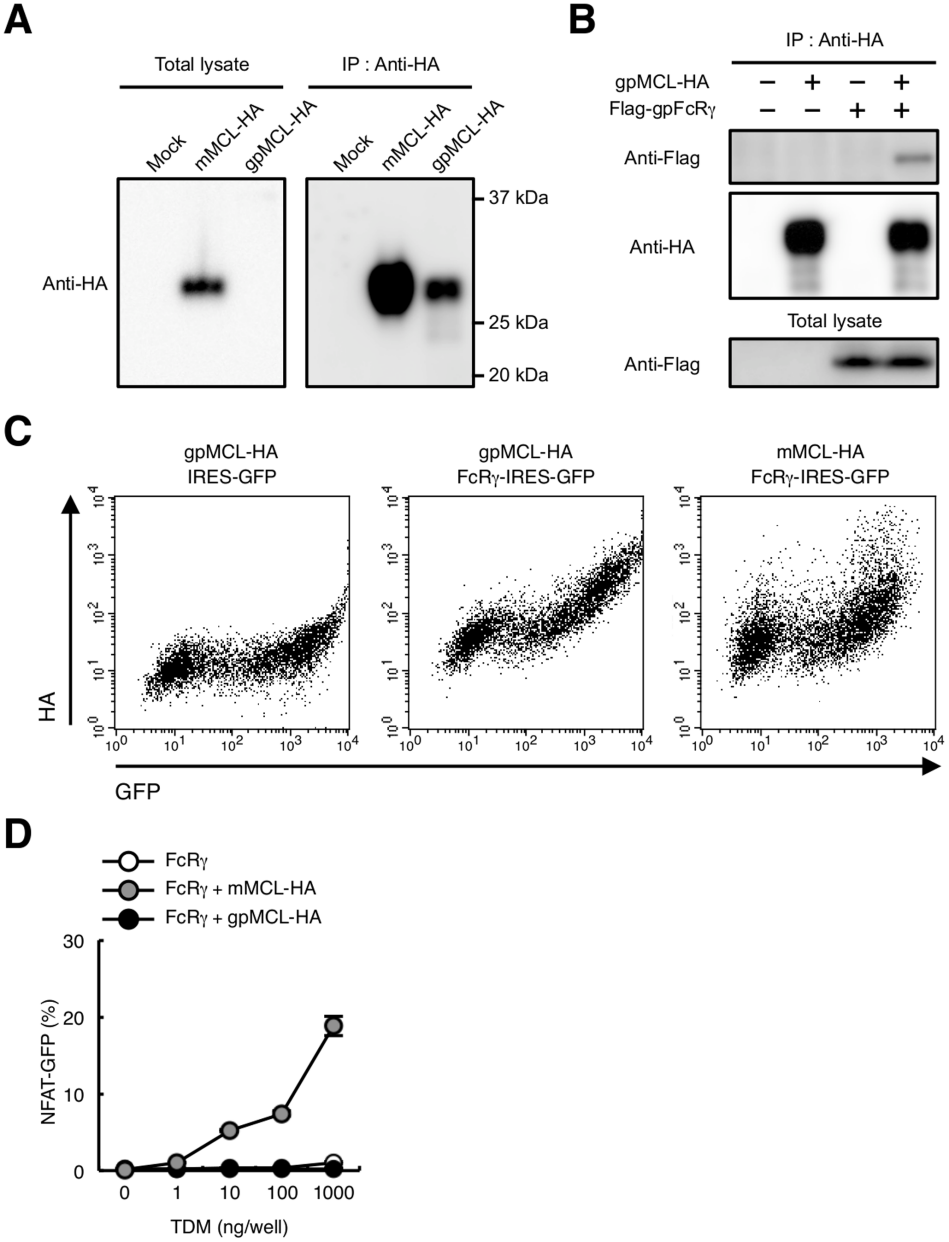
Characterization of gpMCL. (A) Immunoblot of gpMCL. HEK293T cells were transfected with HA-tagged mMCL or gpMCL. Total lysates were blotted with anti-HA mAb (left panel) or immunoprecipitated with anti-HA pAb and blotted with anti-HA mAb (right panel). (B) gpMCL is associated with gpFcRγ. HEK293T cells were transfected with HA-tagged gpMCL alone or together with Flag-tagged gpFcRγ Total lysates were immunoprecipitated with anti-HA pAb and blotted with anti-Flag pAb and anti-HA mAb. Total lysates were also blotted with anti-Flag pAb. (C) Surface expression of gpMCL. HEK293T cells were transfected with HA-tagged gpMCL alone or together with gpFcRγ-IRES-GFP. HEK293T cells were also transfected with HA-tagged mMCL together with mFcRγ-IRES-GFP. Surface expression of gpMCL or mMCL was detected by anti-HA pAb. (D) gpMCL fails to recognize TDM. Indicated reporter cells were stimulated with plate-coated TDM for 24 h. Induction of NFAT-GFP was analyzed by flow cytometry. Data are presented as mean ± s.d. (D) and representative of two or three separate experiments.

To verify whether this truncated protein still retains the MCL function, HA-tagged gpMCL was transfected into the HEK293T cells and total lysates were blotted with anti-HA mAb. Although mMCL was detected at a predicted molecular mass, gpMCL could not be detected (Fig 7A left). Therefore gpMCL was immunoprecipitated using anti-HA mAb. An immunoblotting analysis by anti-HA mAb showed that gpMCL was detected at a predicted molecular mass; however, the signal intensity was significantly weaker compared to mMCL, suggesting that gpMCL may be unstable (Fig. 7A right). This aberrant gpMCL was still associated with FcRγ (Fig. 7B) and expressed on the cell surface in an FcRγ-dependent manner (Fig. 7C).

gpMCL lacks a hydrophobic region that are critical for TDM binding [16]. It was therefore speculated that gpMCL could not recognize TDM. To clarify this possibility, reporter cells expressing gpMCL were established by introducing HA-tagged gpMCL and gpFcRγ into NFAT-GFP reporter cells. TDM failed to activate this reporter cells, as expected (Fig. 7D). These data indicate that MCL does not function as a direct TDM receptor in guinea pigs.

## Discussion

In this paper, we established a monoclonal antibody against gpMincle and demonstrated that gpMincle recognizes TDM and mediates immune responses, including TNF production against mycobacterial infection. Pro-inflammatory cytokine TNF is critically involved in the host defense against mycobacterial infection because TNF-deficient mice showed a decreased survival rate and disrupted granuloma formation following an infection by *M. tuberculosis* [13]. In addition, these mice are highly susceptible for *M. tuberculosis* infection [22], [23]. It was also reported that blockage of TNF by neutralizing antibody attenuated *M. tuberculosis* H37Ra growth in guinea pig macrophages [24]. We showed that macrophages from guinea pigs produced a large amount of TNF in response to TDM and BCG, and these TNF productions were significantly inhibited by newly established anti-gpMincle mAb (Figs. 6B and 6C), suggesting that gpMincle may contribute to the host defense against mycobacteria. *In vivo* use of this antibody may clarify the role of gpMincle in lung granuloma formation and inflammatory responses during mycobacterial infection. In addition, agonistic antibody for gpMincle would be a useful tool as an “antibody-based adjuvant” for the augmentation of the host immunity to protect mycobacterial infection.

Group 1 CD1 proteins that present a mycobacterial lipid antigen to T cells [25], [26] are found in humans and guinea pigs but are lacking in mice and rats [27], [28]. Although it is currently unclear whether group 1 CD1 proteins could present TDM, TDM-related glycolipids have been reported to be presented by CD1 molecules [29], [30]. In addition, TDM potently activates antigen presenting cells (APCs) and elicits T cell responses [7], [8]. For these reasons, it may be possible that TDM or its related glycolipids activate CD1-restricted T cells by two pathways; a direct manner through CD1 molecules and an indirect manner through the activation of APCs *via* Mincle.

We recently reported that both Mincle and MCL are critically involved in TDM responses [7]. Although gpMCL failed to recognize TDM, gpMCL still bound to FcRγ (Figs. 7B and 7C). It was proposed that MCL forms a heteromeric complex with other C-type lectin receptors (CLRs) [31], [32]. Therefore, it would still be possible that gpMCL contributes to mycobacterial responses by forming a complex with other CLRs.

The crystal structure of human Mincle revealed the important regions for TDM binding, such as hydrophobic groove and arginine residue that associate with hydrophobic lipid tail and sugar moiety, respectively [16]. Both of these regions are well conserved in gpMincle, confirming the results of the comparable binding capacity of hMincle and mMincle to bipolar glycolipid, TDM.

Mincle also recognizes the pathogenic fungus *Malassezia spp.* through glycolipids [14], [33]. Guinea pigs were used for the experimental model of *Malassezia*-induced seborrheic dermatitis because symptoms similar to the clinical phenotype are observed on skin infected with *Malassezia spp.* [34]. *Malassezia furfur* and *Malassezia pachydermatis* activated gpMincle reporter cells and anti-gpMincle mAb suppressed this activation (data not shown). Therefore, this antibody is also useful to examine the contribution of gpMincle in *Malassezia*-induced seborrheic dermatitis.

## Conclusion

gpMincle is expressed in lymphoid tissues and myeloid cells including macrophages. gpMincle directly binds to TDM and transduces activation signals through FcRγ. On the other hand, gpMCL lacks functional CRD and fails to bind to TDM. Importantly, gpMincle also recognizes *M. tuberculosis* and *M. bovis* BCG. The monoclonal antibody against gpMincle is established and used to detect the endogenous gpMincle on the activated macrophages. This antibody blocks TNF production upon TDM stimulation in macrophages. TNF production from BCG-infected macrophages is also significantly suppressed by this antibody. Taken together, these observations show that Mincle but not MCL is a direct receptor for TDM in the guinea pig.

## Supporting Information

Figure S1 **Comparison of Mincle and MCL between species.** (A and B) The multiple sequence alignment of Mincle (A) and MCL (B) homologue (m, mouse; r, rat; h, human; gp, guinea pig) by using the ClustalW. The predicted transmembrane domain is underlined. The conserved arginine residue in the transmembrane domain is indicated by filled circle. The EPN motif and WND sequence are shown by black and red box, respectively. Identical and similar amino acid residues are shown in bold and thin asterisks, respectively. Amino acid sequence of gpMCL^2N^ is shown in gray. (C) The alignment of hMCL, gpMCL^2N^ and gpMCL^Ha^. Different nucleotides (g487/c488) between gpMCL^2N^ and gpMCL^Ha^ were shown in red. Stop codon is shown in asterisk. The deduced amino acid sequence of gpMCL^2N^ after the stop codon is shown in gray. Identical and similar amino acid residues between hMCL and gpMCL^2N^ were shown in bold and thin lines, respectively.
(TIF)
