# Supplementary figures and images for: Characterization of the Receptors for Mycobacterial Cord Factor in Guinea Pig
Source: PLoS One. 2014 Feb 12;9(2):e88747. doi: 10.1371/journal.pone.0088747 (PMC3923057; doi:10.1371/journal.pone.0088747)

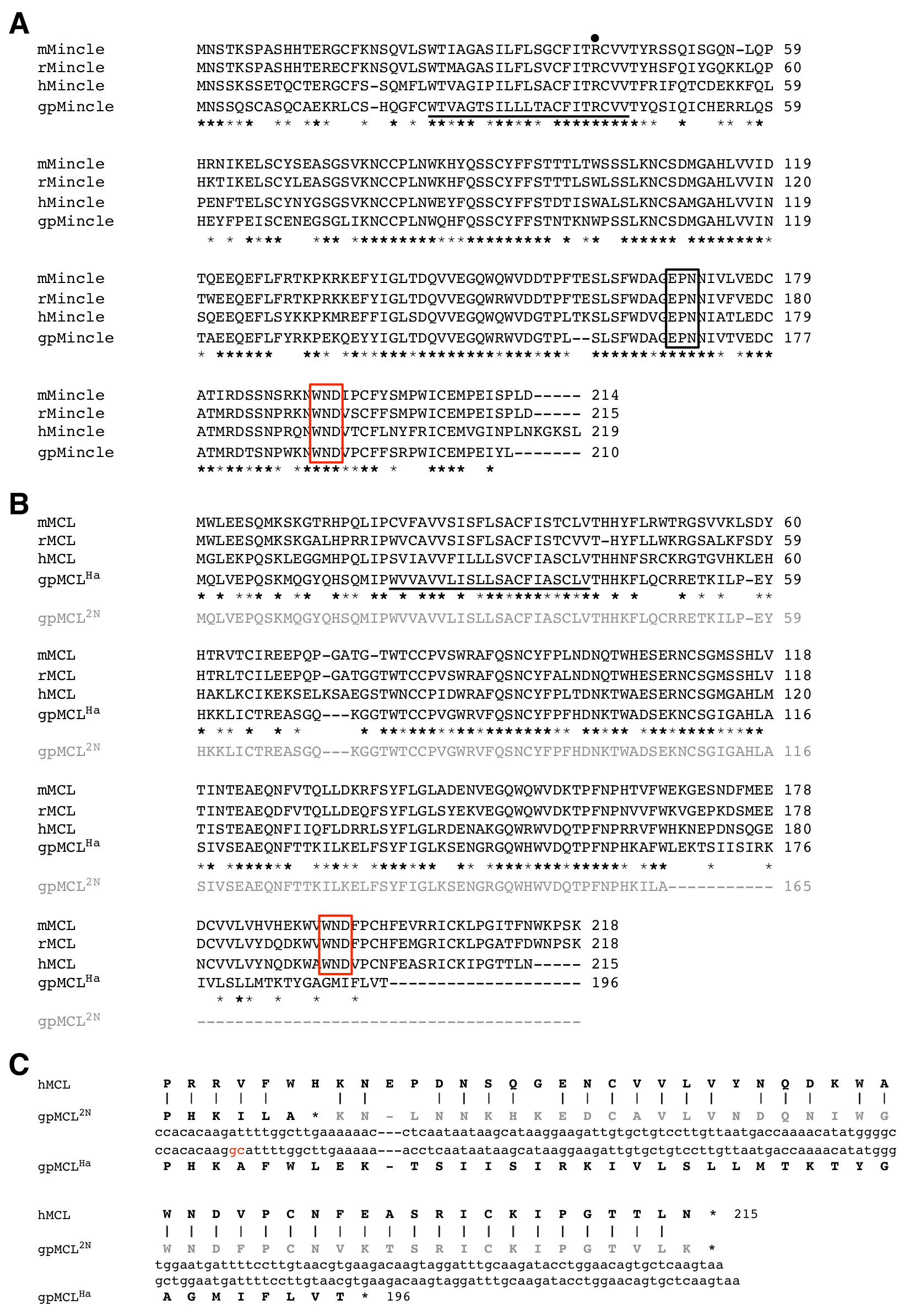

Supplement: Figure S1 — Comparison of Mincle and MCL between species. (A and B) The multiple sequence alignment of Mincle (A) and MCL (B) homologue (m, mouse; r, rat; h, human; gp, guinea pig) by using the ClustalW. The predicted transmembrane domain is underlined. The conserved arginine residue in the transmembrane domain is indicated by filled circle. The EPN motif and WND sequence are shown by black and red box, respectively. Identical and similar amino acid residues are shown in bold and thin asterisks, respectively. Amino acid sequence of gpMCL2N is shown in gray. (C) The alignment of hMCL, gpMCL2N and gpMCLHa. Different nucleotides (g487/c488) between gpMCL2N and gpMCLHa were shown in red. Stop codon is shown in asterisk. The deduced amino acid sequence of gpMCL2N after the stop codon is shown in gray. Identical and similar amino acid residues between hMCL and gpMCL2N were shown in bold and thin lines, respectively. (TIF) [file pone.0088747.s001.tif]
